# Psychosocial perspectives in the treatment of pediatric chronic pain

**DOI:** 10.1186/1546-0096-10-15

**Published:** 2012-06-07

**Authors:** Bryan D Carter, Brooke M Threlkeld

**Affiliations:** 1 Division of Child, Adolescent & Family Psychiatry, University of Louisville School of Medicine, Bingham Clinic, 200 East Chestnut Street, Louisville, KY 40202, USA; 2 Spalding University, 845 South 3rd Street, Louisville, KY 40203, USA

**Keywords:** Chronic pain, Children, Adolescents, Psychosocial, Cognitive behavioral therapy

## Abstract

Chronic pain in children and adolescents is associated with major disruption to developmental experiences crucial to personal adjustment, quality of life, academic, vocational and social success. Caring for these patients involves understanding cognitive, affective, social and family dynamic factors associated with persistent pain syndromes. Evaluation and treatment necessitate a comprehensive multimodal approach including psychological and behavioral interventions that maximize return to more developmentally appropriate physical, academic and social activities. This article will provide an overview of major psychosocial factors impacting on pediatric pain and disability, propose an explanatory model for conceptualizing the development and maintenance of pain and functional disability in medically difficult-to-explain pain syndromes, and review representative evidence-based cognitive behavioral and systemic treatment approaches for improving functioning in this pediatric population.

## Review

### Background

It has been estimated that fifteen to thirty percent of children and adolescents experience chronic pain, with prevalence increasing with age and occurring slightly more commonly in girls than boys [1,2]. Pain is considered a chronic condition when it has persisted for at least three months, moving beyond simple tissue damage (nociceptive) to subsequent changes within the peripheral and central nervous systems (neuropathic). The experience of chronic pain is also impacted by psychosocial factors (stress, negative affective states, family response, etc.) in addition to these biological factors [3]. The most commonly reported locations of pain in children and adolescents are the head, stomach, arms and legs. The most common chronic pain conditions in children include migraine, recurrent abdominal pain, and general musculoskeletal pain [2]. Symptoms of pediatric chronic pain can be severe and disabling, impairing the daily functioning of children and having an adverse impact on their families. Therefore, it is paramount that these chronic conditions be accurately assessed and treated in order to improve functioning and prevent long-term sequelae and deviation from a normal developmental trajectory [3,4].

**Figure 1:**
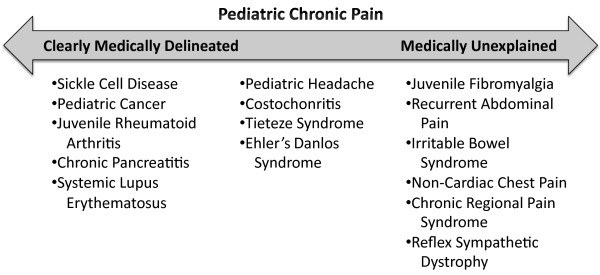
Continuum of painful conditions.

There are a wide variety of medically difficult-to-explain chronic painful conditions that present in the pediatric population, which are often referred to as medically unexplained syndromes (MUS) [5]. These include such conditions as juvenile fibromyalgia syndrome (JFMS), chronic fatigue syndrome (CFS), widespread pain syndrome (WPS), chronic pelvic pain, irritable bowel syndrome (IBS), recurrent abdominal pain (RAP), tension headaches, noncardiac chest pain, complex regional pain syndrome (CRPS), reflex sympathetic dystrophy (RSD), and postural orthostatic tachycardia syndrome (POTS), among others. Central to these conditions is a wide discrepancy in the actual tissue damage sustained by a patient, the perceived severity of the condition, and the degree of disability exhibited. Alternatively, patients with more clearly delineated medical disorders, e.g., childhood leukemias (e.g., ALL, AML), juvenile rheumatoid arthritis (JRA), sickle cell disease (SCD), systemic lupus erythematosus (SLE), to mention some of the major illnesses, often experience limitations in their daily functioning due to the effects of debilitating pain and accompanying fatigue and/or the side effects of treatment. Lastly, there are those painful pediatric conditions which do not fall neatly on either end of the continuum with regard to how well an organic explanation fits the patient’s reported level of pain or disability, e.g., certain headache variants, costochondritis, Ehlers-Danlos syndrome, Tietze syndrome, to give some examples (see Figure 1). The current review will focus primarily on psychosocial approaches to the treatment of the medically difficult-to-explain chronic painful conditions.

In medically unexplained pain syndromes (MUS) it has been suggested that the pain, fatigue, and functional limitations associated with these conditions may be due, at least in part, to abnormalities in centrally-mediated processing functions rather than damage or inflammation of peripheral structures [5]. Neuroanatomically, the hypothalamic-pituitary-adrenal (HPA) axis is often implicated in this process. This dysregulation, which may be triggered by infection, injury or intense physical or psychological stress, results in a malfunction of the central mechanisms that regulate pain, energy, sleep, concentration, memory, etc. [6]. More specifically, increased neural activity in the posterior insula region of the brain has been implicated as at least a component of the sensory amplification seen in these patients [7]. Other central nervous system mechanisms that may be involved in the generation and maintenance of chronic pain include loss of descending analgesic activity and central sensitization, diminished activity of the descending serotonergic-noradrenergic system, and increased activity of endogenous opioidergic systems [7]. Behaviorally, evidence suggests that pain and fatigue can become classically conditioned to (associated with) certain stimuli in the environment, resulting in functional limitations when the individual is confronted with these stimuli. A number of functional imaging studies have demonstrated increased neural activity in brain structures involved in the processing of sensation, movement, cognition and emotion in patients with these conditions, as compared to healthy controls experiencing the same stimuli [8].

Patients with these conditions often experience considerable skepticism and avoidance by health care providers due, in part, to the difficulty in assigning an accurate diagnosis. On the other hand, for the treating physician the patient’s clinical presentation may be frustrating due to the absence of a clear cut etiological explanation, inconclusive investigative tests, and lack of well validated medical treatments. This can lead the physician to ascribe the patient’s symptoms primarily to psychological factors. However, referral to a child psychologist or psychiatrist is often met with defensiveness and even anger on the part of the patient and/or family, who continue to place a high value on finding a specific and readily remediable physical explanation. In such circumstances the patient continues to experience a lowered quality of life with reduced functioning in multiple arenas critical to optimal development. This prolonged frustration of seeking a clear medical explanation and treatment approach may further contribute to symptom exacerbation and feelings of hopelessness. This can set into motion an increased interdependency on parents and caregivers and reduced demands on the patient, that may establish a level of adaptation below the patient’s premorbid baseline level. Secondary gain associated with the sick role and reduced physical activity leading to deconditioning produces further disability [9].

### Psychosocial factors in pediatric pain

A biopsychosocial conceptualization of chronic pain posits a conceptual shift away from attempting to differentiate physical from mental or emotional pain. This model acknowledges the multidimensional nature of pain in which biological, psychological, individual, social and environmental variables are interactive in the development, maintenance, and subjective experience of pain and disability [10-15].

**Figure 2:**
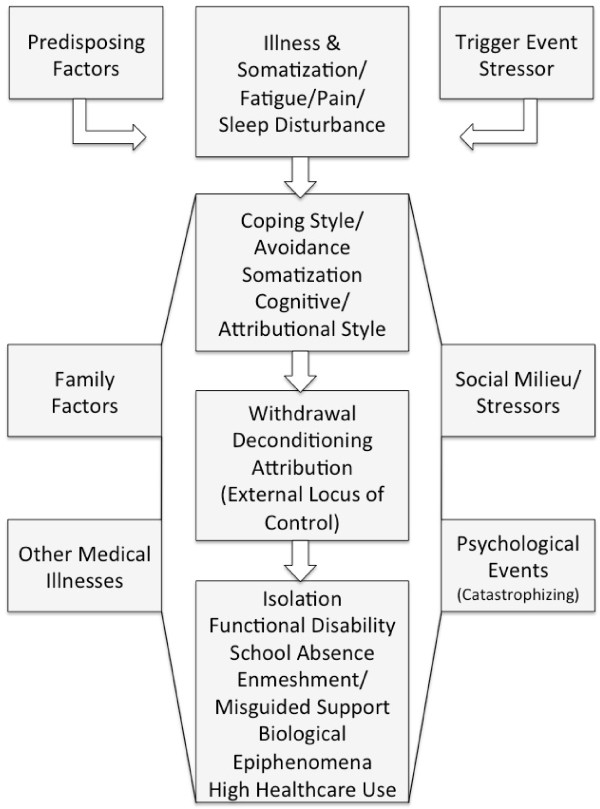
Explanatory model of chronic pain.

A proposed dynamic-interactive explanatory model underlying this process is presented in Figure 2. This model postulates that individuals who develop these conditions may have *Predisposing Factors* or vulnerabilities which, when combined with overt or covert *Trigger Events* (viral illness, psychological stress), result in an initial *Illness* with associated *Somatic Symptoms* including *Pain and/or Fatigue and Sleep Disturbance*. This may lead to the patient engaging in *Avoidance* of activity as a primary *Coping Style*, *Somatization* (increased vigilance to physical symptoms) and *Attribution* of the illness primarily toward events outside their control, i.e., *External Locus of Control*. Influenced by additional events such as *Family Factors* (overprotection, triangulation), *Social Milieu* and *Stressor* factors (peer relationship issues), *Other Medical Illness* (low grade infections, pain/fatigue exacerbations), and *Psychological Events* (catastrophizing cognitions), the patient may engage in further *Withdrawal*, leading to *Deconditioning* and snowballing *Increasing Attributions* that their illness/debilitation is due solely to a unitary disease process for which there is a unitary passive treatment (medication, surgery, etc.). The final pathological outcome is a patient who experiences increasing social *Isolation*, increased *Functional Disability*, *Enmeshment* with their primary caregivers, and additional *Biological Epiphenomena* and *High Healthcare Use* that further reinforce a somatic focus and an emphasis on finding a unitary medical “cure.” The following section will address the findings of investigations supporting components of this explanatory model.

#### Family factors

Chronic pain has an adverse impact on the overall quality of life for both the child and the family [16], leading many to endorse a stress appraisal and coping model in which child disability and family dynamics are a function of the child and family interpretation of symptoms, type of coping employed and parental supportive efforts [9,14,17,18]. For some families the child’s pain symptoms may play a functional role within family interaction patterns and relationships [19-23]. More solicitous parental responses to pain behaviors (e.g., caregivers’ sympathy, attention to symptoms, emotional reactions, modeling of symptoms, and reinforcement of pain behavior via avoidance of responsibilities) have been associated with greater pain, sick role behavior and functional disability, independent of stress level or pain intensity [24-34]. In a study of children with chronic functional abdominal pain, when parents attended to symptom complaints, their children exhibited nearly twice as many such complaints [35]. Table1 gives examples of family dynamics that are frequently observed in patients with chronic pain.

**Table 1:** Family Dynamics Interacting with Pain and Functioning

**Family Dynamic**	**Description**
Overprotection	Belief that restricting patient from activity is needed for improvement in symptoms.
Misguided support	Parental behaviors that either lower expectations or apply excessive pressure for rapid change and improvement in function.
Dysfunctional Communication Patterns	Poor conflict resolution, difficulty communicating affect and avoidance of discussing emotionally charged issues.
Externalized Attributions	Patient and family attribute both illness and recovery solely to factors outside themselves and often beyond their control.
Dependence	Loss of self-confidence due to family members increasingly taking over patient responsibilities and providing assistance that may not necessarily be needed.
Social Isolation and Avoidance	Patient becomes anxious and avoidant of normal peer situations.

#### Coping, cognitive style and personality factors

For children suffering from painful medical conditions, the level at which they are able to function is influenced by a number of individual and systemic factors including child and family interpretation of pain symptoms, type of coping employed, and parental attempts to support their child’s efforts to cope with the pain [15,17,18]. Children with chronic pain have been found to possess too few and ineffective coping strategies and portray themselves as having a lack of control over many aspects of their symptoms [36,37]. Claar, Walker and Smith [38] found pain and functional disability to be more common in pediatric patients with lower perceived competence in academic, social and athletic arenas, and pain-related disability to be reinforced if it allowed the individual to avoid activities at which the child believes him/herself to be ineffective or unsuccessful. Additionally, these children exhibited a tendency towards perfectionism and setting exceedingly high expectations [38]. In contrast, children who employ more active coping strategies report a greater sense of control and display less pain behavior, social withdrawal and functional disability [39,40]. Negative emotions (anxiety, depression) and poor emotional regulation have been found to be associated with greater functional disability [7,41-44]. Furthermore, regardless of the perceived pain severity, when children regard their pain to be a serious health-threatening condition and their coping ability to be low, their pain tolerance is lower.

Children with chronic pain have been observed to report greater pain behavior if they exhibit a cognitive pattern of “catastrophizing,” i.e., a cognitive style characterized by increased focusing on pain and exaggerated/fearful appraisals of pain symptoms and their consequences. This cognitive style is associated with increased pain severity, lower pain tolerance, greater functional disability, more anxiety and depression, and increased use of analgesics [45,46]. The presence of learning disabilities, unrealistic goals in a high-achieving perfectionistic child, early pain experiences, a passive or dependent coping style, marital problems in the home, and chronic illness in a parent have been shown to be associated with visceral pain-associated disability [47]. Clinically, patients experiencing significant functional disability associated with pain have been found to manifest some of the following qualities: extreme conscientiousness, obsessiveness, sensitivity and insecurity; anxious with a tendency to set high standards of achievement resulting in placing considerable stress on themselves; family members who may tend to impose high academic and behavioral standards [11,48].

#### Sleep

Sleep disturbance is prominent in pediatric patients with chronic conditions, particularly when pain is involved [49-55]. As a result, resolution of sleep disturbances plays an important role in the recovery process [50]. The relationship between pain and sleep is bidirectional, i.e., insufficient or disrupted sleep may increase the level of pain experienced and chronic pain increases the likelihood of sleep disruption [56]. While the mechanism responsible for this relationship is not clearly understood, it may be due to a disrupting effect on emotional regulation, attention and behavioral control which impairs the teen’s ability to distract themselves from the pain sensations [56], or to neurophysiological changes that increase pain sensitivity [57,58]. Disturbances in sleep in children have been shown to be a major contributor to increased pain intensity, decreased health-related quality of life, and decreased functioning [51,52,59].

### Psychosocial treatment components and approaches

Psychosocial treatments for chronic pediatric pain attempt to address both symptom reduction and management as well as reduce functional disability and improve quality of life. Such interventions are most effective when employed as part of a multidisciplinary and interprofessional team approach that encourages and facilitates open communication between all health care providers and educators, including the primary care physician, medical specialists, physical therapists, teachers, counselors, etc., in order to orchestrate a return to more normal and developmentally appropriate activities. One or more of these specific components may be needed in combination with pharmacological and physical exercise/therapy interventions. The particular individualized approach employed will vary dependent upon such factors as patient history and symptom presentation, response to previous treatments, unique family dynamics, and environmental factors (service availability, insurance coverage, etc.). Such a comprehensive multidisciplinary approach should be strongly considered when symptoms have become protracted and interfere in a major way with the patient’s ability to function academically, socially or vocationally. The following is a review of specific psychosocial intervention components that have been developed and applied to these conditions. These have applicability to both child and adolescent patient populations.

#### Cognitive behavioral therapy (CBT)

CBT is the most well validated non-pharmacological treatment for chronic pain in pediatric patients [60]. One of the primary goals of CBT is to identify and correct cognitive distortions, which, in the case of chronic pediatric pain, may involve patient and parental beliefs about the child’s illness and such factors as activity restriction, illness exacerbation, school attendance and social involvement, etc., that may impede movement towards rehabilitation. CBT approaches to pediatric pain have been shown to alter patient symptom-related beliefs (attributions) and, subsequently, level of functional disability [61]. Since it has been demonstrated that negative affective states are associated with pain perception and level of functional impairment [42], CBT interventions have also targeted reductions in anxiety, depressive and somatic symptoms. In particular catastrophic thinking, a prime target of CBT interventions, has been associated with poorer outcome and greater functional disability in patients with chronic pain. Some of the major cognitive distortions targeted in treating chronic pediatric pain from a CBT approach are described in Table2. When delivered in a family-based treatment approach, CBT targets parental responses to the child’s pain behaviors, leading to a decrease in reinforcement of these behaviors and increased reinforcement of improved child functioning. Several recent outcome studies of psychosocial interventions with pediatric chronic pain, most involving a strong CBT component, are summarized in Table3.

**Table 2:** Illness-Related Cognitive Distortions Targeted in Cognitive Behavioral Therapy

**Child Cognitive Distortions**	**Parent Cognitive Distortions**
Belief that one must restrict activity and involvement until symptoms resolve	Hesitation to promote increased activity and independence
Self-image and confidence in interpersonal relationships	Fear of worsening patient symptoms by encouraging increased function
Acceptability of assertive communication and behavior	Fear of teen relapse leading to overprotection and overreaction to even minor symptoms
Over-emphasis on peer acceptance and “pleasing others”	Belief that patient is “faking” or exaggerating symptoms
Over-dependence on others in order to recover from illness	Future projection of teen failure due to illness
Setting realistic standards for achievement and success	
Overcoming feelings of invalidation	
Pessimism regarding future health outcome and personal success	

**Table 3:** Recent Intervention Studies for Pediatric Chronic Pain

**Authors (Year)**	**Condition (s)**	**Subjects**	**Modality**	**Outcome**
Kashikar-Zuck, Ting, Arnold, Bean, Powers, Graham, Passo, Schikler, Hashkes, Spalding, Lynch-Jordan, Banez, Richards, & Lovell (2011)	Juvenile Fibromyalgia	114	8 weekly sessions of CBT or FE (Fibromyalgia Education) + 2 “booster sessions”	Significant reduction in functional disability, pain and depressive symptoms with CBT showing significantly greater reduction in functional disability
		11–18 year olds		
Robins, Smith, Glutting, & Bishop (2005)	Recurrent abdominal pain	69	5, 40 min sessions of CBT family intervention with standard medical care (n =40) or standard medical care alone (n = 29)	CBT group reported significantly reduced pain and fewer school absences; no significant between group differences in functional disability or somatization
		6–16 year olds		
Palermo, Wilson, Peters, Lewandowski, & Somhegyi (2009)	Chronic headache, abdominal pain, or musculoskeletal pain	48	8 week, internet-delivered family CBT with sleep and activity interventions and wait-list control with medical care only	CBT group significant reduction in activity limitation and pain post -treatment and 3 -month follow-up. No group difference in depressive symptoms or parental protectiveness.

		11–17 year olds			
Hechler, Blankenburg Dobe, Kosfelder, Hubner & Zernikow (2010)	Chronic, debilitating pain not responding to primary care treatment	33	3 week, multimodal inpatient pain treatment including C BT (individual, family, and group-based), physical therapy, art therapy, medications, and academic support	Significant reduction in pain, disability, school absence, and pain-related coping maintained for 12 coping maintained for 12 months post-treatment	
		7–10 years olds and			
		167			
		11–18 years olds			
Vlieger, Menko-Frankenhuis, Wolfkamp,Tromp & Benninga (2007)	Functional abdominal pain or Irritable Bowel Syndrome	52	6, 50-min sessions of Hypnotherapy (n = 27) or standard medical care with attention/supportive therapy control (n = 25)	Hypnotherapy group reported a greater significant reduction in pain diary ratings of pain intensity and frequency	
		8–18 year olds			
Wicksell, Melin, Lekander & Olsson (2009)	Headache, back/neck pain, Complex Regional Pain Syndrome, and widespread musculoskeletal pain	18	10 weekly sessions of ACT (n = 18) or “Multidisciplinary treatment” with amitriptyline (n = 18)	Greater improvement in ACT group as evidenced by: decreased functional disability, pain intensity, fear of re-injury, and pain interference	
		10–18 year olds			
Scharff, Marcus & Masek (2002)	Migraine headache	36 7–17 year olds	4, 60 min sessions of biofeedback and stress management training with home practice (n = 13) or biofeedback placebo control (n = 11) or waitlist control (n = 12)	Biofeedback and stress management group self reported greater post treatment reduction in migraine pain	

In a study of thirty adolescents with JFMS randomly assigned to 8 weeks of either CBT or self-monitoring, those receiving CBT demonstrated significantly greater ability to cope with pain than those in the self-monitoring only condition [62]. On follow up, subjects had significantly lower levels of functional disability and depressive symptoms compared to baseline, but those who received self-monitoring followed by CBT received the most benefit.

Kashikar-Zuck and colleagues [63] investigated the efficacy of a CBT intervention compared with a fibromyalgia educational (FE) program, for reducing functional disability, pain, and depressive symptoms in 114 adolescents (aged 11 to 18 years) with fibromyalgia. Subjects received usual medical care for eight weeks, and then were randomized to eight weekly CBT or FE sessions with two “booster” sessions. Baseline, eighth week of treatment, and six-month follow-up outcome measures were obtained. The investigators found that almost 88% of participants completed the treatment protocol. Patients from both groups had significantly reduced functional disability, pain, and depressive symptoms. However, CBT was significantly better than FE for reducing functional disability. Both groups had a clinically significant reduction in depressive symptoms, and by the end of the study, the mean scores were in the non-depressed range. However, neither group attained a clinically significant reduction in pain.

#### Acceptance and commitment therapy (ACT)

ACT, is a specific cognitive behavioral approach targeting belief systems in patients suffering from chronic and recurrent painful conditions, has been found to be beneficial for improving perceived functional ability, pain intensity, fear of re-injury, pain interference, and general quality of life [64-66]. ACT differs from traditional CBT approaches to pain management in that the patient learns to accept pain and abandon a primary focus on alleviating pain and the related negative sensations and experiences with which it is associated. Instead the emphasis of treatment is on promoting engagement in meaningful and valued experiences and life involvements. This is accomplished via identifying and gradually exposing the child to valued activities such as attending school, participating in recreational activities and spending time with friends. Acceptance interventions utilize developmentally appropriate mindfulness exercises, which assist with accepting unpleasant internal experiences. Treatment may also involve cognitive externalizing interventions, e.g., characterizing painful sensations as a “pain monster,” in order to separate the experience of pain as independent from one’s global sense of self [67]. ACT appears to improve patient “willingness to function” in spite of pain, anxiety and the potential negative physical consequences of pain.

#### Hypnosis

Clinical hypnosis has a long history as a valuable treatment component with children with chronic pain [68,69], with neuroimaging studies demonstrating its efficacy in activating brain functions known to be involved in the processing of the pain experience [70]. Such hypnotherapeutic interventions as visual imagery, hypnoanesthesia, distancing and distraction techniques, and reframing likely alter the child’s experiencing and interpretation of the sensations associated with pain. A controlled clinical trial of hypnotherapy demonstrated efficacy over standard medical care with supportive therapy in reducing pain intensity and frequency in a group of 8 to 18 year olds with recurrent abdominal pain or irritable bowel syndrome [71].

#### Graduated exercise and activity

Increased physical activity and graduated exercise (GE) are a critical component to recovery from pediatric conditions involving functional disability and pain [72]. A decrease in expectations for physical activity and associated responsibility has been associated with less favorable five-year outcome in adolescents with chronic fatigue and pain [73], and adolescents with significant functional disability associated with their fatigue and pain have been found to have higher levels of parental restriction from activity than their peers with juvenile arthritis [74].

Graduated exercise serves to reverse the effects of physical deconditioning experienced by many children and initiates the process of “re-regulation,” via desensitization of the fear of the physical consequences of overexertion and learning to tolerate temporary discomfort in exchange for later improvement. In addition, increased activity enhances self-confidence regarding one’s ability to function physically and socially and serves to combat the forced dependency imposed by many conditions. Most GE interventions for chronic pain focus on stretching, mobility and aerobic tolerance [75]. Garralda and Chandler [76] have described GE combined with CBT as the most promising intervention for managing painful and fatiguing conditions in adolescents. GE has been included in a number of integrative treatment programs and is valuable in improving debilitating symptoms, sense of “wellness” and school attendance over supportive care alone [77].

#### Improving academic attendance and functioning

Pediatric pain typically has a significant negative impact on academic attendance and performance, though patients will vary in the level of social and academic impairment exhibited. In a study of 221 adolescents with chronic pain, subjects missed an average of 4.5 school days per month and almost half reported a decrease in grades since the onset of their pain condition [78]. Long-term follow-up studies have shown that poorer academic performance and attendance can have lasting negative consequences on an adolescent’s engagement in college-level higher education and employment [79].

Active efforts at school reintegration should be a central component in the treatment of pediatric chronic pain. School systems vary widely in their receptiveness to recommended accommodations and interventions. Therefore, it is important to become familiar with the appropriate federal and state regulations and statutes that apply to pediatric pain conditions, such as the Individuals with Disabilities Education Act (IDEA) and Section 504 of the Rehabilitation Act and the Americans with Disabilities Act, which require accommodations and modifications be made so that the student can fully participate in the classroom and maximally benefit from educational instruction.

#### Sleep intervention

Resolution of sleep disturbances plays an important role in the treatment of chronic pediatric pain. Sleep disturbance alone predicts lower health-related quality of life and higher functional disability in children and adolescents with chronic pain [80]. For example, compared to a no-treatment control group, pediatric patients receiving sleep hygiene education reported a reduction in frequency and duration of migraine headache [81]. Cognitive-behavioral interventions for sleep have also been shown to improve symptoms and quality of life in adolescents with juvenile fibromyalgia [50]. Intervention for sleep involves identifying poor sleep hygiene patterns and implementing strategies to improve sleep regulation. Such strategies include: establishing a regular bedtime routine and sleep schedule, eliminating or limiting daytime naps, limiting caffeine intake, increasing morning sun exposure, discontinuing potentially stimulating activities such as television/computer/video games, etc., within 30 min of bedtime, establishing a healthy morning exercise routine, altering sleep environment to be a cool, dark, quiet and relaxing space that is used exclusively for sleep. Weekly sleep logs are often utilized to record gradual shifts in sleep patterns and to monitor and encourage improved sleep hygiene and quality.

#### Integrated approaches

In a meta-analytic review of twenty-five psychological intervention trials with children and adolescents with chronic pain, Palermo and colleagues [82] found a large positive effect of psychological intervention on pain reduction at immediate post-treatment and follow-up. These studies included youth with headache, abdominal pain and fibromyalgia. Less robust effects were found for improvements in disability and emotional functioning. CBT, relaxation training, and clinical biofeedback all produced significant pain reduction, and this was true for self-administered as well as therapist-administered interventions.

A number of treatment studies have integrated a combination of interventions that include CBT, behavioral, family–systems and GE components. The STAIRway to Health program (Structured Tailored Incremental Rehabilitation) incorporates several aspects of previously researched approaches [75]. Patients and parents are educated in a holistic understanding of their illness that discourages an exclusively physical or psychological approach to understanding the illness. Educational components include explaining vicious cycles that exacerbate illness, including those of nutrition, sleep patterns, physical deconditioning, social isolation, educational estrangement, and emotional cycles (including loss of self-esteem and confidence), as well as bolstering adaptive coping strategies and re-evaluating negative attributions about the prognosis of their illness and the future. A tailored gradual return to school is planned, as well as a gradual return to normal social activity. Though sample size was somewhat small, compared to a control “pacing program” group, the adolescents in the STAIRway group demonstrated significant improvements in school attendance, activity scores, and global health ratings by both the teen and treating clinician. Contrary to common patient and parental precautions to refrain from more vigorous and sustained physical activity, closely monitored active rehabilitation did not exacerbate symptoms in subjects [75].

#### Children’s health and illness recovery program (CHIRP)

**Figure 3:**
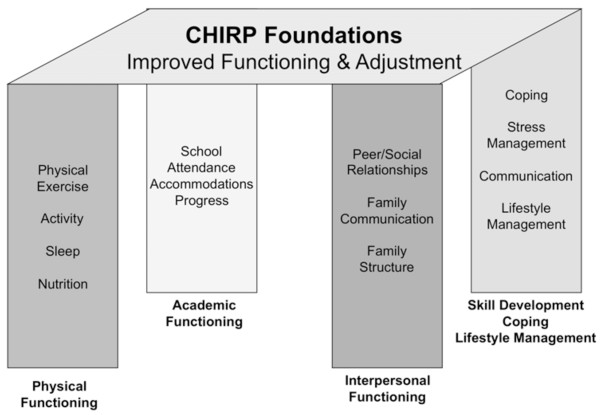
CHIRP treatment model.

The Children’s Health and Illness Recovery Program (CHIRP) is a comprehensive, multimodal outpatient intervention designed for adolescents with chronic painful and fatiguing conditions (Carter, Kronenberger, Bowersox, Hartley, Sherman: Children’s health and illness recovery program clinician’s handbook, unpublished). CHIRP employs a 12-session manualized treatment protocol integrating many validated treatment components in a workbook format, including family-based CBT. CHIRP activities are designed to assist patients in multiple areas including: 1) medical treatment adherence and related lifestyle modifications, 2) identifying and managing stress, 3) improving active coping, time management and problem-solving skills, 4) improving assertive communication and interpersonal relationships, 5) utilizing relaxation and self-soothing behaviors, 6) enhancing functional independence and communication in the context of the patient’s family, and 7) advocating for appropriate educational accommodations. The CHIRP model (see Figure 3) encourages regular communication among patient, family, health care providers and school officials, to facilitate return to school and more normal social functioning. Preliminary clinical outcome data on subjects who completed all sessions demonstrated significant parent- and patient-based ratings of improvement in functioning and reduced pain and fatigue in adolescents with painful and fatiguing illnesses [83].

#### Inpatient treatment for pediatric chronic pain

Multidisciplinary inpatient treatment programs designed to initiate more typical childhood functioning in patients with chronic pain may be indicated for more severe cases that are non-responsive to outpatient interventions. A study of 200 children and adolescents with chronic pain demonstrated the benefits of inpatient multimodal treatment [84]. A multidisciplinary team of pediatricians, clinical psychologists, psychiatrists, nurses, physiotherapists, occupational therapists, and social workers conducted the three-week inpatient treatment. Behavioral health treatment consisted of individual, family, and group therapy sessions incorporating cognitive behavioral principles and trauma-specific interventions. Adjunctive physical therapy, art therapy and medications were also provided. Participants received educational services during the day to promote school reintegration. Three-month and 12-month post-treatment assessment revealed a significant decline in pain intensity, pain-related disability, school absence, and pain-related coping. Participants reported a 60% reduction in use of analgesics at one-year post treatment [84]. The rarity and high cost of such programs reduces accessibility for most patients and may not be appropriate for those who are less impaired and higher functioning. Multimodal outpatient treatment options may be more suitable for these children.

## Conclusions

Comprehensive treatments for pediatric chronic pain need to include attention to psychosocial factors that are associated with and perpetuate symptom persistence and contribute to functional morbidity. There is a growing body of evidence-based literature supporting interventions based upon cognitive, behavioral, family- and social-systems models in improving patient symptoms, quality of life and overall functioning. Future studies need to further investigate these treatments compared to more standard medical interventions alone, as well as efforts to make these interventions more accessible via such mechanisms as digital and web-based tools [85].

## Abbreviations

JFMS, juvenile fibromyalgia syndrome; CFS, chronic fatigue syndrome; WPS, widespread pain syndrome; IBS, irritable bowel syndrome; RAP, recurrent abdominal pain; CRPS, complex regional pain syndrome; RSD, reflex sympathetic dystrophy; POTS, postural orthostatic tachycardia syndrome; ALL, acute lymphocytic leukemia; AML, acute myeloid leukemia; JRA, juvenile rheumatoid arthritis; SCD, sickle cell disease; SLE, systemic lupus erythematosus; MUS, medically unexplained pain syndromes; HPA, hypothalamic-pituitary-adrenal; PADS, pain-associated disability syndrome; CBT, cognitive-behavioral therapy; FE, fibromyalgia educational; ACT, acceptance and commitment therapy; GE, graduated exercise; IDEA, Individuals with Disabilities Education Act; STAIRway, Structured Tailored Incremental Rehabilitation; CHIRP, Children’s Health and Illness Recovery Program.

## Competing interests

The authors declare that they have no competing interests.

## Authors’ contributions

BC literature review and manuscript preparation. BT literature review and manuscript preparation. Both authors read and approved the final manuscript.
